# Ecological continuity in sustainable higher education under SDG 4.7: a process model of participation competence

**DOI:** 10.3389/fpsyg.2026.1821569

**Published:** 2026-04-15

**Authors:** Mengdi Wang, Yanan Guo

**Affiliations:** 1 Department of Foreign Language Teaching and Research, Hebei Normal University, Shijiazhuang, China; 2 Faculty of Educational Sciences Teaching, Xingtai University, Xingtai, China

**Keywords:** classroom ecology, ecological continuity, education for sustainable development (ESD), institutional support, oral language agency, sustainable competencies

## Abstract

**Purpose:**

Grounded in the framework of Sustainable Development Goal 4. 7 (SDG 4.7) and Education for Sustainable Development (ESD), this study focuses on the core issue of “sustained participation and cross-contextual continuity” as a key sustainable competence in higher education. It aims to construct a process-oriented mechanism model explaining how this competence is generated, internalized, and institutionally sustained within classroom ecology. Taking classroom participation practices as an interactive field, the study explores how sustainable competence is constructed and maintained within multi-actor interaction structures and institutional conditions.

**Methods:**

A qualitative research design was adopted. Using an interaction-oriented course as the research context, semi-structured interviews were conducted with students, teachers, and organizational/institutional support representatives (*N* = 29). Guided by constructivist grounded theory, the study employed open coding, axial coding, and selective coding (three-level coding) for systematic integration. Triangulation across multiple participant perspectives was further applied to enhance analytical rigor and interpretive robustness.

**Findings:**

Four interrelated mechanisms were identified: (1) The reconstruction of the participation risk framework, through which classroom interaction shifted from a high-exposure structure to a low-risk participation structure; (2) The institutionalization and internalization of teacher interaction rules and peer collaboration norms, transforming participation from a high-cost individual risk-taking behavior into a low-cost shared routine; (3) The formation of a sustaining strategy system, enabling participation to achieve operational stability and repeatability; and (4) The ecological and institutional continuity conditions required for cross-contextual extension, as well as the sustainability constraints triggered by ecological discontinuities.

**Conclusion:**

This study proposes an integrated mechanism model of “safe generation – agency internalization – ecological continuity,” demonstrating that sustainable participation competence is not an inherent individual trait but an ecological form of competence embedded within interaction structures and institutional design. Its long-term stability depends on the coordinated alignment among instructional rules, collaborative norms, and mechanisms of institutional continuity.

## 1 Introduction

Grounded in the framework of Sustainable Development Goal 4.7 (SDG 4.7) and Education for Sustainable Development (ESD), higher education is regarded as a critical stage for cultivating “sustainable competencies.” Such competencies are not centered primarily on disciplinary knowledge itself; rather, they emphasize cross-contextual continuity, sustained participation, collaborative engagement, and self-regulation, as well as the capacity to cope with uncertainty in complex social environments (Ha and Nguyen, 2021). From this theoretical perspective, “sustainability” in education is no longer measured primarily by short-term performance improvement or stage-based achievement. Instead, it focuses on whether learners are able to sustain participation and maintain agency across diverse contexts. When this concept is situated within interactive classroom settings, sustainable competence is manifested in whether learners can continuously engage in interaction or meaning negotiation within socially embedded learning environments, and manage their perceived participation risks in uncertain or evaluative situations. However, existing research indicates that classroom silence and avoidance do not stem from a lack of ability, but are closely related to learners' social interpretations of error, evaluation, social consequences, and relational structures (Wakelin et al., 2024). In interactive environments, mistakes may be interpreted as socially threatening, thereby significantly increasing the psychological cost of participation and inhibiting sustained engagement (Li and Fan, 2026). Therefore, the central challenge of sustainable education does not lie in merely improving task performance, but in how to institutionalize interactional conditions that reduce participation risk and support sustained engagement. From the perspective of classroom ecology, sustained participation should not be understood as the result of individual willpower, but as the outcome of the coordinated functioning of multi-layered support structures. This includes how teachers formalize interaction norms through instructional design and rule arrangement, how peers form supportive participation structures in collaborative practice, and whether institutional arrangements can provide conditions of continuity beyond the classroom (Holst et al., 2024). On this basis, sustainable competence is not an inherent attribute possessed by individuals, but an ecological construct embedded within interaction structures and institutional conditions. It is important to note that this study does not equate classroom participation *per se* with “sustainability.” Rather, it conceptualizes sustainable participation competence as a process-oriented capacity through which learners sustain engagement, regulate participation risk under conditions of social evaluation, and achieve cross-contextual continuity.

Although research on sustainability-oriented education has continued to increase in recent years, existing studies have predominantly focused on the identification and classification of sustainability competencies, with comparatively limited attention to the process through which such competencies are enacted and sustained within interactional contexts (Cebrián et al., 2020; Hugo-Van Dyk et al., 2023; Redman and Wiek, 2021). Against this background, the present study situates an interaction-oriented course context within the framework of a sustainable learning ecology, proposing that the classroom can be understood as an ecosystem jointly constituted by instructional rules, interaction practices, and institutional resources. Unlike research orientations centered on disciplinary skill acquisition or stage-based outcomes, this study focuses on how sustainable participation competence is generated and stabilized within structured interaction environments, and why such competence may encounter ecological discontinuities beyond the classroom. Furthermore, grounded in ESD theory, this study proposes a cyclical mechanism model of “safe generation – agency internalization – ecological continuity” to provide a process-oriented explanation of how sustainable competence is generated within interaction structures, internalized through the integration of norms and strategies, and extended across contexts under conditions of institutionalized continuity. Accordingly, the following research questions are proposed:

RQ1: how do learners reinterpret and reconstruct their perceived participation risks in interaction-oriented educational contexts? How does this process influence their participation patterns and sustained engagement?
RQ2: how are teacher-designed interaction rules and peer collaboration norms internalized by learners and transformed into observable manifestations of *sustainable participation competence*?
RQ3: under what ecological and institutional conditions can participation competence constructed in the classroom be sustained in broader practice fields? Under what conditions do ecological discontinuities occur?

In this study, *sustainable participation competence* is used as a superordinate concept referring to the competence structure through which learners maintain sustained engagement and achieve cross-contextual extension within socially interactive contexts. In oral interactive classrooms, this competence is manifested in contextualized form as *oral language agency*. The model of *safe generation—agency internalization—ecological continuity* is used to describe the mechanism chain of its generation and extension, in which “ecological continuity” specifically refers to the institutionalized conditions of continuity between classroom ecology and broader practice ecology.

## 2 Theoretical framework

### 2.1 ESD, SDG 4.7, and the ecological orientation of sustainable competencies

Within the policy framework of the United Nations SDG 4.7, higher education is entrusted with the important mission of cultivating “sustainable competencies” (King et al., 2025; Šimić Šašić and Atlaga, 2024). Unlike educational models oriented toward stage-based achievement or single-instance performance, Education for Sustainable Development (ESD) emphasizes the development of cross-contextual continuity of competence in authentic social contexts, including sustained participation, collaborative communication, risk management, self-regulation, and the capacity to cope with uncertainty (Gonda et al., 2024). This theoretical orientation indicates that sustainable competence is not the result of a single learning episode, but a form of competence that is gradually generated and maintained within interaction practices and institutional support structures (Brundiers et al., 2021; Nguyen et al., 2024). Compared with outcome-oriented evaluation logics, ESD places greater emphasis on the learning process itself and the interaction structures and institutional conditions in which it is embedded, highlighting the central role of multi-actor collaboration and supportive environments in the sustained development of competence (Bohm et al., 2024). From this perspective, the “sustainability” of learning is reflected in whether learners are able to sustain participation in complex and uncertain situations, rather than in short-term performance improvement (Metelski et al., 2021). Accordingly, this study conceptualizes the classroom as a learning ecology jointly constituted by instructional rules, interaction practices, and institutional resources, and defines “sustainable participation competence” as a process-oriented competence structure that is embedded in context, constructed through social interaction, and maintained through ecological continuity. The theoretical significance of classroom participation in this study lies not in its role as an isolated classroom behavior, but in its manifestation of learners' capacity to sustain action, regulate participation risk under conditions of uncertainty, and achieve cross-contextual continuity.

### 2.2 Participation risk and safe generation

In interaction-oriented educational contexts, the foundation of sustained participation does not lie in skill proficiency itself, but in how learners interpret and manage their perceived participation risks. Existing research indicates that classroom silence and avoidance do not stem from a lack of ability, but are closely related to learners' social interpretations of error, evaluation, social consequences, and relational structures (Lindqvist and Forsberg, 2023). In highly evaluative interaction environments, mistakes may be interpreted as socially threatening, thereby significantly increasing the psychological cost of participation and limiting sustained engagement (Busch et al., 2023). From the perspectives of social interaction theory and situated learning theory, risk is not an inherent attribute, but an institutional outcome constructed through classroom rules, feedback logics, and interaction scripts. When evaluation structures and error-correction approaches change, learners' interpretive frameworks of risk also adjust accordingly (Günther et al., 2022). Within the framework of sustainable competence, “safe generation” constitutes the foundational mechanism of sustained participation (Gautam et al., 2025). Only when participation is redefined as a practice activity that can be accommodated and supported, and when risk is institutionally reduced, can learners enter a stable interaction process. Therefore, this study regards the reconstruction of the risk framework as the starting point for the generation of sustainable participation competence.

### 2.3 Interaction rules, peer norms, and agency internalization

Within the classroom ecology, teachers do not merely perform the function of knowledge transmission, but construct institutional conditions for “how to participate” through rule design and interaction organization (Wei and Cao, 2024). Feedback approaches, evaluation pacing, and interaction governance logics directly influence the social cost and safety structure of participation. Compared with interaction models characterized by immediate evaluation and high correction density, rule arrangements that emphasize meaning negotiation, process continuity, and clearly defined tolerance boundaries help reduce the risk of participation interruption, thereby making sustained interaction a legitimate and predictable practice pathway (Li et al., 2024). In addition, peer interaction constitutes another important institutional support mechanism. Through collaborative communication, emotional responsiveness, and shared responsibility, learners redistribute participation risks within interaction and maintain engagement motivation (Huang et al., 2024). When these interaction practices are collectively shared and stabilized, participation is no longer an individual risk-taking behavior, but an institutionalized routine within the classroom ecology. Within the ESD framework, this stabilization of rules and norms enables participation competence to shift from external support to internal regulation, achieving “agency internalization” (Schlunegger et al., 2024). Sustainable participation competence does not originate from individual willpower, but is gradually generated within institutionalized interaction structures and internalized into stable participation patterns. This mechanism constitutes the core component of the “safe generation – agency internalization” cyclical structure.

### 2.4 Sustaining strategy systems

After “rules–norms” become institutionalized, learners obtain the feasibility and predictability of participation; however, what is “feasible” does not automatically mean “sustainable.” Sustainability further depends on whether learners develop a set of micro-regulatory resources that can be repeatedly mobilized, enabling them to maintain interaction continuity under conditions of uncertainty and imperfection. Existing research indicates that action sustainability often relies on the integration of resources such as cognitive pre-organization, emotional regulation, and situational self-regulation, rather than on a single structural support (Bohm et al., 2024; Xu et al., 2022). Under conditions of structural support, learners gradually develop alternative action pathways, time-regulation strategies, and meaning-prioritized task advancement patterns, allowing interaction to continue under imperfect conditions (Wilkins et al., 2022). Therefore, a sustaining strategy system is not a collection of fragmented techniques, but an integrated and routinized set of regulatory resources formed through practice. It constitutes the operational bridge through which “agency internalization” is transformed into “stable practice,” enabling sustained participation to achieve repeatability at the level of action.

### 2.5 Ecological discontinuity and institutional continuity

Although relatively stable participation practices may be formed within the classroom, existing research indicates that learning transfer does not occur automatically, and learners often face significant challenges when applying what has been learned in class to new contexts (Huang and Lajoie, 2023). Extracurricular environments generally involve greater uncertainty, weaker support structures, and stronger perceptions of social risk, making it difficult for participation scripts formed in the classroom to be directly extended. Research on cross-contextual activities between “classroom–out-of-class/school” settings has likewise pointed out the complex challenges involved in connecting classroom and out-of-school contexts, emphasizing that learning continuity must be intentionally facilitated rather than naturally occurring (Cattaruzza et al., 2022). From ecological and institutional perspectives, constraints on transfer do not result from insufficient individual effort, but from changes in structural conditions. When resources, time, and organizational support are insufficient, and when institutionalized follow-up mechanisms are lacking, the application of competence is often constrained by contextual conditions, resulting in “easily interrupted” practice outcomes (Flick, 2022). Therefore, the key issue in sustainable education does not lie in short-term performance improvement, but in whether institutionalized mechanisms exist to connect classroom ecology with broader practice ecology. Emphasizing the design of cross-setting “learning ecosystems/social–educational ecologies” has been regarded as an important direction for supporting the sustained development of learning across multiple settings (Johnson et al., 2020). Such “ecological continuity” constitutes the structural condition for the long-term maintenance of competence. Based on the above theoretical dimensions, this study proposes an integrated cyclical mechanism model of “safe generation – agency internalization – ecological continuity” to explain how sustainable participation competence is generated within interaction structures, stabilized through the integration of norms and strategies, and either extended or interrupted under different institutional conditions.

## 3 Methods

### 3.1 . Research design

This study adopted a qualitative research design (Bazen et al., 2021) with the aim of understanding how learners construct their sustainable participation competence in authentic classroom contexts, and of examining the roles played by teacher interaction rules, peer collaboration norms, and organizational/institutional support. The study does not test linear causal relationships among variables; rather, it focuses on learners' experiential interpretations of classroom interaction, participation risk, and sustained engagement, as well as the internal mechanisms underlying their generation. Against the background of ESD's emphasis on long-term competence development and a learning ecology orientation, this study conceptualizes the classroom as a learning ecology jointly constituted by instructional rules, interaction practices, and institutional support. Constructivist grounded theory was adopted as the methodological framework (Lin et al., 2025), as it emphasizes the generation of explanatory theory grounded in specific contexts and enables the analysis of how learners construct participation meanings and their mechanisms of sustained engagement within interactional structures (Bobbink et al., 2024). Through semi-structured interviews, participants were invited to describe their classroom participation experiences, key interaction situations, and their understandings of how participation competence is generated and extended across contexts. In doing so, the study reconstructs the ecological generation logic of sustainable participation competence. The sampling framework, interview protocol, and coding procedures are presented in the Supplementary Materials (see Supplementary Table S1-S3) to ensure transparency and traceability of the research process.

### 3.2 Research participants

This study employed purposive sampling combined with a maximum variation strategy to select participants capable of providing rich experiential descriptions from different role perspectives (Campbell et al., 2020). A total of 29 participants were included, covering three groups: students, teachers, and organizational/environmental support representatives, in order to achieve multi-perspective triangulation of classroom practice, instructional decision-making, and institutional conditions (Sadoughi and Hejazi, 2024). This study was conducted at two comprehensive universities in China. Student participants (*N* = 16) were undergraduate students majoring in Education and Psychology, aged 19–23, with Chinese as their native language and English as a foreign language. Teacher participants (*N* = 8) were English instructors responsible for delivering the course, aged 35–46. Institutional support representatives (*N* = 5) were drawn from relevant teaching and administrative roles, aged 40–52. This participant composition enables a multi-perspective analysis across learners, instructional implementation, and institutional conditions. An overview of participant composition and data sources is presented in Table 1. To ensure anonymity and analytical clarity, a unified coding scheme was adopted: S represents students, T represents teachers, and OR represents organizational/environmental support representatives. All quotations are presented in coded form and do not contain any personally identifiable information. A more detailed sampling framework and participant background information are provided in Supplementary Table S1.

**Table 1:** Overview of research participants and data sources.

Participant category	Number	Heterogeneity coverage and key stratification	Coding rule	Data source
Students	16	Coverage across 4 academic years: Year 1 (4), Year 2 (5), Year 3 (5), Year 4 (2); maximum variation in gender and classroom participation level; majors in education and psychology; aged 19–23; native Chinese speakers with English as a foreign language	S1–S16	Semi-structured in-depth interviews; structured question framework + contextualized probing (event-oriented follow-up questions)
Teachers	8	All fully taught the course; teaching experience: 4–7 years (4), ≥8 years (4); long-term practice of supportive and interaction-oriented teaching; aged 35–46; English language instructors	T1–T8	Same as above
Organizational and environmental support representatives	5	Responsible for or supporting course systems/resources/operations: vice dean for teaching, department chair, director of language learning center, academic affairs administrator, course coordinator; aged 40–52; institutional and administrative roles related to course implementation	OR1–OR5	Same as above

The focal course examined in this study was a 16-week interaction-oriented English speaking course designed for non-native English learners. Instruction was organized through interactive tasks such as group discussions, role plays, and thematic oral expression. The course was guided by the instructional principles of “meaning-first” and “delayed correction.” Classroom evaluation emphasized process-oriented participation, with the aim of reducing participation risk and supporting sustained interaction.

### 3.3 Data collection

Semi-structured interviews were employed as the primary data source in this study. Semi-structured interviews facilitate thematic focus while providing participants with sufficient space to articulate the meanings of their experiences (UNESCO, 2017). The interview design combined a pre-established question framework with contextualized inquiry to ensure systematic coverage of key mechanisms in the classroom learning process, while preserving openness in participants' expressions. Each interview lasted approximately 45 min. With participants' informed consent, interviews were audio-recorded and subsequently transcribed verbatim after completion. The interview content was organized around four core modules (see Supplementary Table S2 for details): (1) Overall course experience and changes in participation; (2) Key classroom situations related to participation risk, interaction safety, feedback approaches, and collaborative practices; (3) Regulatory strategies and action resources adopted by learners during interaction; and (4) Cross-contextual extension of classroom experience and its institutional support conditions.

### 3.4 Data analysis

A three-level coding procedure grounded in constructivist grounded theory was employed to systematically analyze the interview transcripts (Jornet et al., 2025). The analysis followed the constant comparative method to ensure that concepts and categories were progressively refined through data comparison (Maher and King, 2022).

(1) Open coding: the interview transcripts were conceptually annotated line by line, resulting in the extraction of 132 initial concepts (examples are presented in Supplementary Table S3-1).
(2) Axial coding: based on the properties and relationships among concepts, seven main categories were developed, including risk perception construction, supportive interaction norms, utilization of strategy resources, maintenance of expressive fluency, manifestation of agency, contextual dependency of transfer, and institutional support needs (see Supplementary Table S3-2).
(3) Selective coding: these categories were further integrated into four core themes (see Supplementary Table S4-S7), on the basis of which a process model of “safe generation – agency internalization – ecological continuity” was constructed (Supplementary Table S8). This model is used to explain the generation mechanism of oral language agency within classroom ecology and the institutional conditions affecting its extension beyond the classroom.

In addition, to ensure credibility and interpretive rigor, this study followed established quality standards for qualitative research and adopted multiple strategies for assurance (Flick, 2022; James, 2024): (1) Multi-perspective triangulation was implemented across students, teachers, and organizational/environmental support representatives to enhance the robustness of interpretation; (2) Two researchers independently coded approximately 30% of the interview transcripts and reached consensus through discussion to unify conceptual and categorical definitions, thereby improving analytical consistency; (3) Theoretical saturation was determined through constant comparison, and was considered achieved when subsequent interviews did not introduce new core themes; and (4) Member checking was conducted after theme development, and reflective memos were recorded throughout the research process to enhance transparency and traceability of the analysis.

## 4 Findings

This study conducted a systematic analysis of interview transcripts from students, teachers, and organizational/environmental support representatives. Following the three-level coding procedure, 132 initial concepts were integrated into seven axial categories. Analytically, these seven axial categories corresponded to four mechanism domains: risk interpretation, interaction norms, strategy resources, and transfer conditions. During selective coding, they were further integrated into four core themes (see Table 2), jointly supporting the mechanism model of “safe generation – agency internalization – ecological continuity.” The core analytical conclusion running throughout the entire dataset is that sustainable participation agency is not a stable individual trait or a single attitudinal tendency, but a form of sustainable competence embedded within classroom interaction ecology. Around this central interpretive direction, selective coding further integrated four interrelated core themes: (1) Reconstruction of the participation risk framework; (2) Internalization of interaction norms and the emergence of agency; (3) Generation of a sustaining participation strategy chain; and (4) Ecological conditions for cross-contextual sustained participation. At the analytical level, these four themes constitute an interrelated interpretive framework (see Supplementary Materials Tables S3–S8).

**Table 2:** Overview of themes in the sustainable learning ecosystem.

Core theme (selective coding)	Subtheme	Conceptual connotation (analytical definition)
Theme 1: reconstruction of the participation risk framework	Risk perception based on rule uncertainty (rule ambiguity anxiety; anticipated social consequences)	Learners' uncertain interpretations of classroom tolerance boundaries and social evaluative consequences constitute the primary upstream constraints on participation avoidance; “non-participation/limited participation” is understood here as a rational response to social exposure.
	Interaction safety as an alternative participation script (perceived interaction safety)	Non-judgmental responses from teachers and peers provide an observable and imitable participation script, in which imperfect responses are no longer interpreted as social threats but as absorbable learning events.
	Risk tolerance as a shared routine (risk tolerance and composed participation)	When interaction safety is repeatedly practiced and collectively recognized, risk interpretation is transformed into a stable participation expectation, providing a socially tolerable foundation for sustained participation.
Theme 2: internalization of interaction norms and emergence of agency	Teacher-led interaction rule design (delayed judgment; meaning-first principle)	Through institutionalized rules, teachers govern classroom interaction pacing and feedback logics, legitimizing participation continuity as a learning objective and systematically reducing participation cost.
	Formation of peer collaboration norms (peer collaborative compensation)	Within the framework of teacher rules, peers develop collaborative compensatory practices, collectively sharing interaction risk and sustaining participation as a shared norm.
	Agency as an observable participation position (active participation; undertaking challenges)	Sustainable participation agency is manifested as learners' repositioning within interaction—from passive response to actively initiating, managing, and supporting others' participation.
Theme 3: generation of a sustaining participation strategy chain	Language resources as anchors of meaning (meaning organization; emotional buffering)	Learners use available resources (e.g., notes and peer prompts) as functional tools for cognitive organization and emotional regulation, reducing immediate load and entering a participation state.
	Micro-strategies for maintaining participation (interaction maintenance micro-strategies)	Through alternative expression, filler responses, rhythm control, and similar approaches, learners prioritize maintaining interaction continuity rather than pursuing one-time “perfect performance.”
	Strategy routinization and participation focus (strategy integration; interaction orientation)	When strategies are integrated into stable practices, cognitive resources shift from self-monitoring to meaning negotiation and task advancement, enabling agency to achieve self-sustaining capacity at the operational level.
Theme 4: ecological conditions for cross-contextual sustained participation	Contextual activation conditions for transfer/extension (contextual triggers; familiar networks)	Participation practices formed in the classroom are more easily activated in contexts where real-world demands are explicit and social risk is buffered by familiar relationships.
	Risk of ecological discontinuity (ecological discontinuity)	When out-of-class environments lack clear norms, low-risk scripts, and stable support structures, sustained participation practices are difficult to maintain.
	Need for institutionalized linkage (continuity design)	Institutionalized arrangements connecting classroom ecology with broader learning/practice ecology constitute key conditions for supporting the extension of sustainable participation agency.

### 4.1 Theme 1: risk framework reconstruction

The findings of Theme 1 derive from participants' consistent descriptions of “why they were unwilling to participate” and “what an imperfect response signifies.” In open coding, “rule ambiguity anxiety” and “anticipated social consequences” repeatedly emerged as upstream constraining factors. Participation avoidance was not manifested as a lack of ability, but rather as a rational response by learners to potential negative evaluation within highly socially exposed classroom contexts. The analysis indicates that changes in the risk framework were not achieved through abstract shifts in psychological states, but were gradually established through interaction safety as a perceptible and imitable classroom participation script.

#### 4.1.1 Rule uncertainty and risk perception

Regarding rule uncertainty and risk perception, the majority of students (13/16) reported that their avoidance of classroom interaction primarily stemmed from an uncertain understanding of the boundaries of classroom rules. S2 described:

“At the beginning, I didn't dare to speak at all because I didn't know what would happen if I said something wrong… Would the teacher criticize me? Would it affect my usual performance score?”

S7 also mentioned that, due to uncertainty about whether the use of Chinese was permitted as a compensatory response, she chose to remain silent. In addition, “imperfect responses” were widely associated with social consequences such as “losing face” or “being evaluated by peers.” S4 stated:

“What I'm most afraid of is not that the content is not good enough, but that if I speak in a strange way and classmates laugh behind my back, that would be too embarrassing.”

These narratives indicate that participation avoidance in the early stage of the classroom did not originate from learners' competence itself, but from a high-risk interpretation of the classroom social context.

#### 4.1.2 Interaction safety as participation script

Across both student and teacher accounts, a key mechanism repeatedly emerged: the experience of being “allowed to continue” during interaction. Imperfect responses or interruptions did not lead to the termination of interaction or escalation of evaluation; rather, they were absorbed into the interaction process itself. This experience was not an abstract form of “encouragement,” but a repeatedly practiced participation script that demonstrated to learners how interaction should proceed under conditions of imperfect participation. S9 stated:

“The teacher did not interrupt me.”

More importantly, this sense of safety was not confined to the individual level, but was socially distributed through the overall classroom interaction order: peers waited, interaction was not interrupted, and participation continued to advance. S15 noted that the teacher maintained interaction through restatement or rephrasing rather than public judgment:

“The teacher just repeated my idea more clearly and let him continue… there was no criticism.”

These micro-level practices stabilized learners' expectations regarding

“what happens when participation is imperfect.”

#### 4.1.3 Risk tolerance as shared routine

With sustained exposure to such interaction scripts, learners gradually integrated new interpretations of risk into stable participation expectations. S7 described:

“Now in class, I don't first think about whether it is correct; I focus more on whether I have expressed the meaning clearly. If there is a problem, I just add another sentence and move on.”

S11 similarly pointed out that a shared understanding had formed in the classroom:

“Perfection is not the goal; participation itself is.”

Theme 1 indicates that the foundational premise of sustainable participation agency is not the enhancement of motivation, but the redefinition of the risk framework under the support of interaction safety, rendering participation socially tolerable and repeatable. This finding demonstrates that the generation of sustainable participation competence does not originate from the natural growth of individual self-confidence, but depends on the institutional reorganization of the classroom risk structure. When “imperfect responses” are redefined as absorbable learning events, participation becomes socially tolerable and repeatable, thereby establishing the structural foundation for the generation of sustainable competence.

### 4.2 Theme 2: norm internalization and agency emergence

Theme 2 demonstrates that when classroom rules and interaction norms are institutionalized and practically enacted by learners, participation can be sustained. In axial coding, “supportive interaction norms” emerged as a key category, closely associated with teacher-led rule design and peer collaborative practices. Agency did not appear here as a form of self-reported attitude, but was manifested in learners' repositioning of their roles within interaction.

#### 4.2.1 Teacher rule design: delayed judgment and meaning priority

Regarding teacher-designed interaction rules, all teachers (8/8) did not conceptualize support as a single attitude, but rather as a set of interaction governance principles. T4 stated:

“Expressing ideas is more important than performing perfectly at once. I do not interrupt because of imperfection; I focus first on communication and progression.”

T7 likewise emphasized maintaining interaction continuity by not interrupting speech:

“I provide prompts or rephrase the question, rather than correcting or judging immediately…”

These accounts indicate that teacher support functions as an institutionalized rule: by enhancing the predictability of classroom interaction and establishing participation continuity as the legitimate objective, it systematically reduces the cost of participation.

#### 4.2.2 Peer norms and collective risk sharing

The significance of peer support does not lie in “friendliness,” but in the formation of a collaborative norm. Under this norm, interaction risk is redistributed to the collective level. Students described compensatory practices, such as helping participants who became stuck to complete their expressions, providing prompts, or maintaining group interaction. S15 stated:

“If someone gets stuck, others will help continue the sentence, like assisting a teammate.”

S9 likewise noted a clear shift in peer support:

“Later, no one laughed at others for speaking unfluently… people would even ask what he wanted to express.”

These practices indicate that peers, as carriers of norms, continuously reinforced the meaning-first participation logic through everyday interaction.

#### 4.2.3 Agency as interaction position

When rules and norms were internalized, learners' behaviors in interaction were repositioned. Agency was manifested in active participation or undertaking challenges, as well as in supporting others' participation. S14 stated:

“Now I will actively challenge more difficult topics because I know failure will not have serious consequences.”

In addition, S9 described assuming the role of norm transmission:

“I will encourage quieter classmates.”

Theme 2 defines sustainable participation agency as an ecologically empowered position: learners are able to act agentically because the participation system itself renders such action feasible. This result further indicates that agency is not a transformation of individual personality or attitude, but an ecological position formed after institutionalized interaction rules and collaborative norms become stabilized. Sustainable competence is therefore manifested as the outcome of an interaction system structure that reduces the cost of action and provides predictable support, rather than as a unilateral strengthening of individual willpower.

### 4.3 Theme 3: sustaining strategy formation

Theme 3 explains how agency is maintained at the micro-action level. The analysis indicates that agency does not rely on a single act of courage, but is stabilized through operational strategies.

#### 4.3.1 Available resources as meaning anchors

Students commonly regarded the use of “available resources” as functional tools for entering a participation state. S5 stated:

“I first organize the key points in Chinese… and then start speaking.”

In addition, S11 mentioned using self-talk for emotional regulation:

“Before speaking, I tell myself ‘don't panic' in my mind.”

In the analysis, such resources were coded as strategic tools that reduce cognitive load and stabilize participation. They do not primarily indicate improvement in language skills, but rather function as “operational entry points” through which participation action can be initiated and sustained.

#### 4.3.2 Micro-strategies for interaction continuity

Regarding sustaining participation strategies, a majority of students (12/16) reported that they tended to prioritize maintaining interaction rather than striving for one-time perfect expression. S14 described:

“If I suddenly don't know how to say something, I immediately switch to a simpler expression to keep the interaction going.”

In addition, S16 pointed out that rhythm control and filler responses were used to gain thinking time. Accordingly, success was redefined as maintaining participation rather than achieving formal accuracy of response. Sustainable participation competence is thus manifested in the capacity to maintain action continuity and interaction progression under conditions of uncertainty and imperfection.

#### 4.3.3 Strategy routinization and participation focus

When these strategies were repeatedly applied and integrated into routine practice, learners described reduced cognitive burden and increased focus on interaction meaning:

“Now these strategies feel natural, and I can focus on the dialogue itself.” (S11)

S7 similarly stated:

“I care more about whether others understand me, rather than small mistakes.”

Theme 3 indicates that agency acquires self-sustaining capacity through the formation of strategies. This demonstrates that the stability of sustainable competence does not depend on a single courageous act, but on a set of operational, integrable, and repeatable strategy systems. Once strategies become routinized, participation shifts from occasional attempts to sustainable practice, enabling agency to obtain structural support at the operational level.

### 4.4 Theme 4: ecological conditions for continuity

Theme 4 directly addresses the issue of sustainability. The analysis indicates that agency generated within classroom ecology does not automatically extend to broader contexts; its continuation is highly dependent on ecological consistency and institutional support. This suggests that the stability of sustainable participation competence is not maintained solely through in-class practice or individual willingness, but depends on whether cross-setting support structures can achieve continuity and accessibility at the institutional level.

#### 4.4.1 Contextual activation and familiar networks

Students described that when real-world demands were explicit and social risk was buffered by familiar networks, participation practices formed in the classroom were more easily activated. S9 stated:

“When I see international students needing help, I will directly use English.”

In addition, S15 mentioned that mixed use of English would occur in familiar environments such as dormitories. These accounts indicate that cross-contextual extension is manifested more as contextual activation than as the automatic output of stable competence: when task meaning is clear and relational networks provide buffering, learners are more likely to bring participation scripts formed in the classroom into everyday practice.

#### 4.4.2 Ecological discontinuity

Regarding ecological discontinuity, a majority of students (11/16) reported that when the support structures established in the classroom could not be reproduced in external contexts, participation was easily interrupted. S15 contrasted the English Corner with classroom experience:

“The English Corner feels too unfamiliar; it's not like our classroom.”

In addition, S14 emphasized the impact of structural absence:

“Without fixed arrangements, it is hard to persist in practice.”

These accounts demonstrate that ecological discontinuity is primarily reflected in the absence of clear rules, predictable feedback, stable peer structures, and sustained participation scenarios, thereby preventing learners from entering a participation state at low cost in new environments.

#### 4.4.3 Institutionalized linkage design

Accounts at the organizational level explicitly defined sustainability as a system design issue. OR1 stated:

“We plan to extend this low-risk model into regular English workshops.”

In addition, OR3 proposed establishing stable speaking partners and thematic spaces. These suggestions collectively indicate that only through institutionalized linkage can classroom ecology align with broader learning and practice settings.

Accordingly, sustainability is not an internally stable trait of learners, but an issue of institutional design. When classroom ecology cannot achieve structural alignment with broader practice settings, participation competence is difficult to sustain. The key to sustainable education lies in constructing institutionalized mechanisms of cross-contextual continuity, rather than merely improving in-class performance or relying on individual self-discipline.

### 4.5 Integrated mechanism model

**Figure 1:**
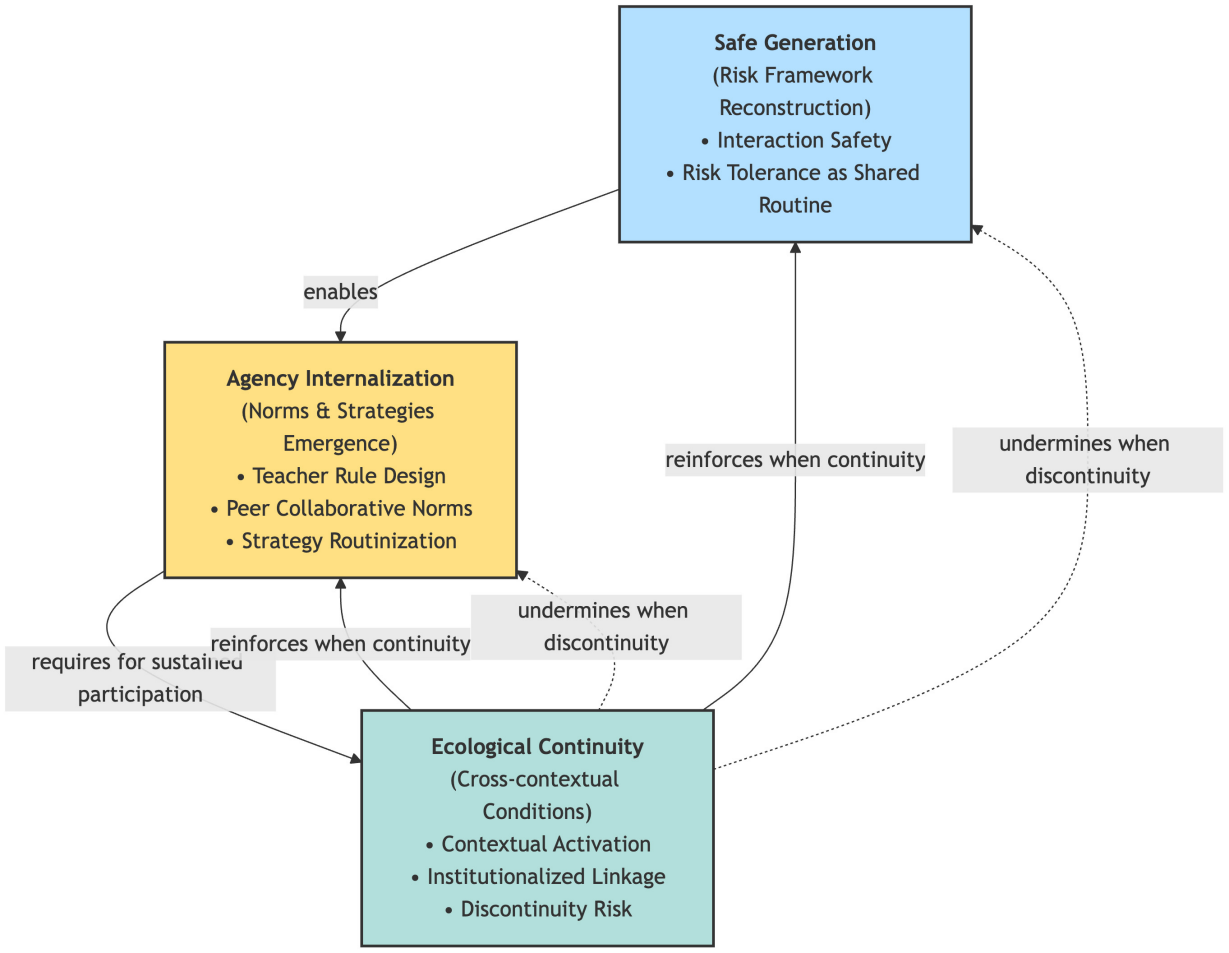
Mechanism model of “safe generation—agency internalization—ecological continuity”.

By integrating the four themes above, the study identified a set of interrelated key mechanisms: (1) The participation risk framework is redefined under the support of interaction safety, making sustainable participation agency socially possible; (2) The internalization of teacher rules and peer norms enables agency to obtain an institutionalized position within interaction; (3) Through the integration of sustaining participation strategies, agency acquires operational stability; and (4) Only under conditions of institutionalized continuity can such agency be maintained across contexts (Figure 1).

Based on these mechanistic relationships, this study proposes an integrated model of “safe generation – agency internalization – ecological continuity.” The model emphasizes that: (1) Agency is not a stable individual trait; (2) Sustainable competence is the product of ecological embeddedness; and (3) The continuation of competence depends on institutionalized linkage design. It is important to note that this model does not describe stages of temporal development, but represents an analytical integration of the mechanism relationships manifested in participants' experiences. It is intended to explain the generation logic of sustainable participation competence within educational ecology and its institutional constraint conditions. Moreover, ecological continuity is not a terminal condition. When institutionalized linkage in out-of-class settings reproduces low-risk scripts and stable collaborative structures, learners' interpretations of “participation risk” are reinforced again, thereby reciprocally consolidating risk tolerance and norm internalization within the classroom. Conversely, ecological discontinuity causes the risk framework to rebound, weakening established strategy routinization and the agentic position. This cyclical relationship indicates that sustainable participation competence is not linearly accumulated, but manifests as an ecological mechanism of dynamic reinforcement or attenuation under different institutional conditions.

## 5 Discussion

Based on multi-actor qualitative data, this study systematically explicates how sustainable participation agency is generated, stabilized, and constrained within classroom ecology. The findings indicate that sustainable participation agency is not a stable internal individual trait, but a form of sustainable competence that depends on interaction rules, social norms, and institutional linkage conditions. Through the reconstruction of the participation risk framework, the internalization of interaction norms, and the integration of strategies, learners' participation behaviors shift from high-risk attempts to repeatable practices. However, its cross-contextual extension does not occur naturally, but is highly dependent on ecological continuity design. This section discusses the findings in relation to RQ1–RQ3 and integrates the four themes into the structural mechanism of “safe generation – agency internalization – ecological continuity.”

### 5.1 Risk framework reconstruction and safe generation

In response to RQ1, the study found that participation risk in interactive classrooms does not stem from a singular deficiency in ability, but from a system of risk interpretation constructed within specific interaction institutions and evaluative structures. Existing research indicates that classroom “mistakes” are often associated with face threat and social evaluation, thereby increasing the psychological cost of participation (Busch et al., 2023; Lindqvist and Forsberg, 2023; Wakelin et al., 2024). Furthermore, risk perception is not a stable trait, but an institutional expectation regarding classroom rules and interaction consequences. When tolerance boundaries and evaluative logics are unclear, participation avoidance becomes a rational choice. This finding elevates classroom silence from the psychological level to the institutional level. Risk is not objectively given, but reproduced within rule uncertainty and evaluative structures. Therefore, the generation of sustainable participation competence depends on the reorganization of the risk structure, rather than on the mere strengthening of motivation. When rules such as the “meaning-first principle” and “delayed judgment/delayed correction” become institutionalized, imperfect responses are redefined as processual phenomena, and interaction continuity gains central importance. This shift in rules reduces social cost and enhances interaction predictability. Ecological systems theory suggests that competence generation depends on structural conditions that redistribute risk and resources (Ha and Nguyen, 2021). The present study verifies this mechanism at the micro-interaction level. From the perspective of ESD and SDG 4.7, sustainable competence implies the capacity for sustained participation in uncertain social contexts (Cebrián et al., 2020). If the classroom is organized as a high-exposure space, competence cannot be stabilized. Accordingly, “safe generation” is essentially a process of institutional reorganization, through which risk is structurally reduced, rendering participation a tolerable and repeatable mode of action.

### 5.2 Peer norm resonance and social regulation

In relation to RQ2, the study found that when teacher interaction rules and peer collaboration norms form a stable interaction order, participation shifts from an individual act of courage to an institutionalized routine. Teachers, by governing interaction pacing and feedback logics, assign central importance to interaction continuity. In addition, peers, through collaborative compensation and risk sharing, transform participation into a shared practice. This finding extends existing research on teacher support and learning motivation (Holst et al., 2024). Traditional models often conceptualize teacher support as an individually perceived variable; however, the present study demonstrates that its core mechanism lies in the institutionalized arrangement of interaction rules. Recent research on embodied teacher support similarly indicates that teacher support promotes sustained participation and student well being through structured interaction practices, further validating the institutionally embedded nature of support (Leal Filho et al., 2019). Support is not an expression of attitude, but a reorganization of the risk structure through rules. Once rules are stabilized, participation is no longer interpreted as a high-risk attempt, but becomes a routine mode of classroom action. Peer norms further reinforce this structure. Social interaction theory suggests that participation behaviors are replicated and maintained through group norms (Gautam et al., 2025; Huang et al., 2024). In the context of this study, peer collaboration shifts risk from the individual level to the collective level, thereby reducing psychological cost and rendering sustainable participation agency an “ecological position” conferred by the interaction structure. From the perspective of sustainable education, multi-actor collaboration constitutes a key condition for the long-term maintenance of competence (Ha and Nguyen, 2021). ESD emphasizes interaction networks within learning ecosystems (Johnson et al., 2020), and the present study concretely demonstrates how rule stabilization and norm internalization transform sustainable participation agency into a sustainable participation pattern.

### 5.3 Internalization of external support

Building on the reconstruction of risk and the stabilization of norms, this study further demonstrates that external support can only be transformed into stable competence through a process of strategy integration. In the classroom, “available resource support” (such as peer prompts, teacher restatement, and task scaffolding) together with “alternative responses/interaction-maintenance strategies” constitute an operational system for sustaining participation, enabling participation to shift from occasional behavior to sustainable practice. Existing research has largely focused on the relationship between strategy use and learning performance (Schlunegger et al., 2024; Wilkins et al., 2022), with relatively limited attention to understanding strategy formation within a sustainable competence framework. The present study indicates that the formation of strategies is a key mechanism for competence stabilization. When sustaining participation strategies are integrated into automatized action resources, learners are able to maintain participation even in contexts lacking immediate external support. This finding responds to ESD's concern with the long-term development of competence (Gonda et al., 2024), demonstrating that sustainable competence is not merely a matter of attitude or value endorsement, but also involves the accumulation and integration of operational action resources. The sustainability of competence is therefore manifested as the result of the coordinated functioning of structural support and strategy resources, rather than as an abstract psychological state.

### 5.4 Ecological conditions of agency transfer

In response to RQ3, the study indicates that sustainable participation agency generated within the classroom does not automatically extend to out-of-class contexts. Transfer is manifested more as contextual activation than as the stable output of competence. Related research likewise shows that language use beyond the classroom is highly dependent on contextual support and social networks, rather than on the natural extension of classroom-based competence (Muhamad Noor et al., 2024). When out-of-class environments fail to reproduce the low-risk scripts, normative clarity, and stable support structures of the classroom, participation behaviors are easily interrupted. This finding challenges the assumption of natural competence transfer (Hugo-Van Dyk et al., 2023) and confirms the ecological theoretical perspective that competence is embedded within specific structural conditions (Sadoughi and Hejazi, 2024). When structural conditions change, the visibility and sustainability of competence change accordingly (Bobbink et al., 2024). The present study illustrates this mechanism of ecological discontinuity within a concrete classroom context. From the policy perspective of SDG 4.7, educational sustainability implies that competence can extend across diverse social contexts (Ha and Nguyen, 2021). However, in the absence of institutionalized linkage design, structural discontinuities arise between classroom ecology and broader learning/practice ecology, causing competence to remain confined within the classroom. Organizational-level suggestions, such as the establishment of regularized practice platforms, stable peer networks, and thematic participation spaces, indicate that sustainability is fundamentally an issue of institutional design rather than insufficient individual effort (Bohm et al., 2024). Accordingly, “ecological continuity” should be regarded as the core condition for the extension of sustainable competence, elevating educational sustainability from the level of individual psychology to the level of systems and institutional design.

### 5.5 Theoretical contributions and implications

This study makes three primary theoretical contributions: (1) At the conceptual level, the study redefines sustainable participation agency as a form of sustainable competence embedded within classroom ecology, rather than as a static motivational or attitudinal variable. By explicating the relationships among risk framework reconstruction, stabilization of interaction norms, and integration of strategy resources, the study demonstrates that sustainable participation agency is a process-oriented competence generated under structural conditions of support; (2) At the mechanistic level, the study identifies the synergistic pathways among teacher rule design, peer collaboration norms, and strategy integration, clarifying how sustainable participation competence becomes institutionalized within multi-actor interaction and achieves operational stability; and (3) At the model level, the study proposes the integrated framework of “safe generation – agency internalization – ecological continuity,” systematically explaining why participation competence generated in the classroom does not naturally extend across contexts. The framework positions sustainability as an issue of institutional continuity rather than as a simple extension of individual effort. Accordingly, through a mechanistic explanation, this study incorporates sustainable participation agency into the theoretical framework of sustainable education, providing a structural perspective for understanding the ecological conditions of competence generation and extension.

### 5.6 Limitations and future research directions

Although this study examined the construction mechanism and ecological constraints of oral language agency through multi-perspective qualitative analysis, several limitations remain; (1) This study adopted a cross-sectional qualitative design. The analysis was based on participants' holistic descriptions of classroom experience and did not track real-time changes in learning behavior throughout the course implementation process; (2) This study focuses on an interaction-oriented English course situated within a specific institutional context. Therefore, the findings are more appropriately interpreted as an analytical explanation applicable to similar interactive learning environments, rather than as direct generalizations to broader educational settings. Their applicability across different disciplinary, linguistic, and cultural contexts requires further examination. Future research may build upon the present study by adopting longitudinal tracking or mixed-methods designs to more systematically investigate the operational mechanisms of oral language agency across different learning ecologies. In addition, future studies may examine how institutionalized linkage design functions across diverse educational contexts, thereby further enriching understanding of sustainable language learning ecology.

## 6 Conclusion

Grounded in the frameworks of ESD and SDG 4.7, this study systematically presents the generation mechanism of sustainable participation agency within classroom ecology and the institutional constraints affecting its cross-contextual extension through constructivist grounded theory analysis. The findings indicate that sustainable participation agency is not an inherent individual attribute, but a form of ecological competence embedded within interaction rules, peer norms, and institutional conditions. (1) Sustainable participation begins with the reconstruction of the participation risk framework. When interaction rules such as the “meaning-first principle” and “delayed judgment/delayed correction” become institutionalized, participation risk is redefined and interaction continuity gains central importance. Learners' participation shifts from high-exposure attempts to tolerable and repeatable practices, thereby acquiring a structural foundation of safety; (2) Under conditions in which teacher rules and peer norms are stabilized, participation transforms from an individual act of courage into an institutionalized routine, and agency becomes an ecological position conferred by the interaction structure. Participation no longer depends on the strengthening of individual willpower, but is supported and maintained within predictable institutional arrangements; and (3) Through the integration of classroom resource support and a sustaining strategy system, learners transform external support into operational action resources, enabling participation to acquire self-sustaining capacity at the operational level. This process of strategy integration shifts participation from occasional behavior to sustainable practice, providing a micro-level foundation for competence stabilization. However, agency generated within the classroom does not naturally transfer to broader practice settings. In the absence of institutionalized linkage design, structural discontinuities arise between classroom ecology and wider social practice environments, limiting the extension of competence. The development of sustainable educational competence therefore depends on ecological continuity rather than on the mere enhancement of in-class performance. Based on these mechanisms, this study proposes the integrated model of “safe generation – agency internalization – ecological continuity,” defining sustainable competence as an ecologically co-constructed process among learners, pedagogical practices, and institutional support. The model elevates educational sustainability from the level of individual psychology to the level of interaction structures and institutional design, providing a mechanistic explanatory framework for the generation, maintenance, and cross-contextual extension of sustainable competence in higher education.

## Data Availability

The original contributions presented in the study are included in the article/Supplementary material, further inquiries can be directed to the corresponding author.
