# Supplementary material for: Ecological continuity in sustainable higher education under SDG 4.7: a process model of participation competence
Source: Front Psychol. 2026 Apr 15;17:1821569. doi: 10.3389/fpsyg.2026.1821569 (PMC13124924; doi:10.3389/fpsyg.2026.1821569)
Supplement: Supplementary file 1 [file Table_1.DOCX]

**Supplementary Table S1. Participant Overview and Data Sources**

| **Participant Category** | **n** | **Heterogeneity Coverage and Key Stratification** | **Coding Scheme** | **Data Sources** |
| --- | --- | --- | --- | --- |
| Undergraduate Students | 16 | Coverage across four academic years: Year 1 (4), Year 2 (5), Year 3 (5), Year 4 (2); maximum variation in gender and classroom participation level; majors in Education and Psychology; aged 19–23; native Chinese speakers with English as a foreign language | S1–S16 | One-time semi-structured in-depth interviews; structured question framework with contextualized probing (mechanism-oriented follow-up questions) |
| Course Instructors | 8 | All fully taught the course; teaching experience: 4–7 years (4), ≥8 years (4); long-term practice of supportive and interaction-oriented teaching; aged 35–46; English language instructors | T1–T8 | Same as above |
| Organizational/Institutional Support Representatives | 5 | Responsible for or supporting course systems/resources/operations: Vice Dean for Teaching, Department Chair, Director of Language Learning Center, Academic Affairs Administrator, Course Coordinator; aged 40–52; institutional and administrative roles related to course implementation | OR1–OR5 | Same as above |
| Total | 29 | Three-role triangulation across “classroom – instruction – institutional support” perspectives | 29 unique identifiers | 29 verbatim interview transcripts |

**Supplementary Table S2. Semi-Structured Interview Protocol**

| **Interview Module & Analytical Purpose** | **Student Questions** | **Instructor Questions** | **Institutional Questions** | **Rationale and Analytical Use** |
| --- | --- | --- | --- | --- |
| Module 1: Participation Risk and Evaluation Logic (Identifying risk framework) | What are the main risks of participating? What rules increase or reduce hesitation? | How do you define acceptable mistakes? How do you manage evaluation pressure? | What institutional arrangements influence participation climate? | → Generates data for risk framework reconstruction (Theme 1) |
| Module 2: Interaction Safety and Rule Implementation (Capturing low-risk scripts) | Describe an interaction where you felt “allowed to continue.” What reactions felt safe/unsafe? | Provide a classroom example reflecting your interaction philosophy. How do you maintain continuity? | — | → Produces thick description of interaction safety, delayed correction, peer collaboration (Themes 1–2) |
| Module 3: Sustaining Strategies and Resource Use (Identifying strategy chains) | How do you maintain participation under difficulty? What resources help you? | What strategies do students use? How do you balance support and autonomy? | — | → Generates data for strategy integration and operational stabilization (Theme 3) |
| Module 4: Cross-Contextual Extension (Identifying ecological conditions) | When are you more likely to participate beyond class? What limits it? | What is the most sustainable outcome of this course? What institutional design supports continuity? | What mechanisms provide stable participation platforms? | → Directly informs ecological continuity and institutional linkage (Theme 4 & model) |

***Note:*** *Questions are designed to elicit mechanism explanations rather than stage-based narratives*

**Supplementary Table S3. Coding Procedure Overview**

**Table S3-0. Initial Concept Codes and Core Theme Mapping**

| **Initial Code** | **Core Theme**  **(S4–S7)** | **Operational Definition** |
| --- | --- | --- |
| A1 Rule Ambiguity Anxiety | Theme 1 | Risk amplification due to unclear tolerance boundaries |
| A2 Anticipated Social Consequences | Theme 1 | Linking mistakes to face threat or identity risk |
| A3 Perceived Interaction Safety | Theme 1 | Experience of acceptance and uninterrupted participation |
| B1 Delayed Judgment / Meaning Priority | Theme 2 | Prioritizing meaning continuity over immediate correction |
| C1 Resource Anchoring | Theme 3 | Use of available resources for cognitive or emotional regulation |
| D1 Contextual Activation | Theme 4 | Participation triggered by situational demand |
| E1 Institutional Continuity Need | Theme 4 | Expectation for stable platforms and structural linkage |

**Table S3-1. Open Coding Examples**

| **Initial Concept** | **Definition** | **Representative Quote** | **Participant** | **Analytical Note** |
| --- | --- | --- | --- | --- |
| Rule Ambiguity Anxiety | Uncertainty about participation norms | “I don’t know where the boundary is, so I choose not to speak.” | S2 | Evidence for Theme 1 |
| Interaction Safety | Mistakes absorbed without interruption | “The teacher did not interrupt me.” | S9 | Key mechanism |
| Delayed Correction | Meaning prioritized over grammar | “I don’t interrupt because of mistakes.” | T4 | Institutional rule |
| Resource Anchoring | Resource use to stabilize participation | “I organize my thoughts first before speaking.” | S5 | Strategy initiation |
| Institutional Continuity Need | Need for stable participation platforms | “We need a fixed group.” | S15 | Sustainability gap |

**Table S3-2. Axial Coding: Seven Main Categories**

| **Axial Category** | **Aggregated Concepts** | **Theoretical Orientation** |
| --- | --- | --- |
| Social Construction of Risk | A1, A2 | Participation constrained by evaluation logic |
| Supportive Interaction Norms | A3, B1 | Risk reshaped by institutionalized scripts |
| Resource Instrumentalization | C1 | Learners manage cognitive and emotional load |
| Interaction Continuity Orientation | Alternative expression,  pacing control | Success redefined as continuity |
| Operationalized Agency | Active initiation, challenge-taking | Agency as observable position |
| Context-Dependent Extension | D1 | Participation activated by context |
| Institutional Continuity Need | E1 | Sustainability depends on structural linkage |

**Table S3-3. Selective Coding: Integration into Four Core Themes**

| **Core Theme** | **Integrated Categories** | **Explanatory Contribution** |
| --- | --- | --- |
| Theme 1 Risk Framework Reconstruction | Social risk → Supportive norms | Explains how risk becomes socially tolerable |
| Theme 2 Norm Internalization and Agency Emergence | Rules + Peer norms → Active participation | Explains how agency becomes institutionalized |
| Theme 3 Sustaining Strategy Formation | Resource use → Strategy integration | Explains operational stabilization |
| Theme 4 Ecological Conditions for Continuity | Agency + Context + Institution | Explains extension constraints and activation |

**Supplementary Table S4. Theme 1: Risk Framework Reconstruction**

| **Subtheme** | **Representative Quote** | **Participant** | **Mechanism** |
| --- | --- | --- | --- |
| Pre-existing Risk Frame | “I don’t know what will happen if I’m wrong.” | S2 | Rule uncertainty amplifies risk |
|  | “I’m afraid of being laughed at.” | S4 | Mistake framed as identity threat |
| Interaction Safety Script | “The teacher said ‘take your time’ and let me continue.” | S9 | Low-risk script established |
| Risk Tolerance Normalization | “Perfection is not the goal.” | S11 | Risk reinterpreted as routine |

**Supplementary Table S5. Theme 2: Norm Internalization and Agency Emergence**

| Subtheme | Representative Quote | Participant | Mechanism |
| --- | --- | --- | --- |
| Teacher Rule Governance | “We finish communication first.” | T4 | Institutionalization of meaning priority |
| Peer Risk Sharing | “We help each other continue.” | S15 | Collective redistribution of risk |
| Emergent Agency | “I now choose challenging topics.” | S14 | Reduced cost expands action space |

**Supplementary Table S6. Theme 3: Sustaining Strategy Formation**

| **Subtheme** | **Representative Quote** | **Participant** | **Mechanism** |
| --- | --- | --- | --- |
| Resource Anchoring | “I organize my ideas first.” | S5 | Cognitive pre-structuring |
| Interaction Continuity | “I switch to simpler words.” | S14 | Continuity prioritized over accuracy |
| Strategy Routinization | “These strategies feel natural.” | S11 | Automatization reduces monitoring |

***Note:*** *Organized by functional mechanism rather than developmental stages*

**Supplementary Table S7. Theme 4: Ecological Conditions for Continuity**

| **Subtheme** | **Representative Quote** | **Participant** | **Mechanism** |
| --- | --- | --- | --- |
| Contextual Activation | “When someone needs help, I participate.” | S9 | Demand triggers agency |
| Ecological Discontinuity | “The setting feels unfamiliar.” | S15 | Lack of structural alignment |
| Institutional Linkage | “We will expand this model institutionally.” | OR1 | Sustainability as design issue |

**Supplementary Table S8. Integrated Ecological Model of Sustainable Participation Competence**

| **Logical Component (Non-Temporal)** | **Core Process** | **Key Mechanisms** | **Evidence Chain** | **Contribution to**  **SDG 4 / ESD** |
| --- | --- | --- | --- | --- |
| Component A | Low-Risk Interaction Ecology | Meaning-first rule, delayed judgment, safety script | S2 → S9 → S7 | Inclusive participation (SDG 4.5) |
| Component B | Operationalized Agency | Norm internalization, strategy integration | T4 → S15 → S11 | Learner empowerment (SDG 4.7) |
| Component C | Ecological Continuity | Context activation + Institutional linkage | S9 → OR1 | System alignment for sustainability |

*Integrated Model: Safe Generation – Agency Internalization – Ecological Continuity Instructional Rules × Learner Strategies × Institutional Support*

***Note:*** *Components represent analytical constructs rather than developmental stages*
